# Anesthetic Effect of the Fascia Iliaca Compartment Block with Different Approaches on Total Hip Arthroplasty and Its Effect on Postoperative Cognitive Dysfunction and Inflammation

**DOI:** 10.3389/fsurg.2022.898243

**Published:** 2022-05-04

**Authors:** Tengchen Feng, Jibo Zhao, Jiayi Wang, Xiaojia Sun, Tong Jia, Fulong Li

**Affiliations:** Department of Anesthesiology, The First Affiliated Hospital of Hebei North University, Zhangjiakou, Hebei, China

**Keywords:** fascia iliaca compartment block, total hip arthroplasty, postoperative cognitive dysfunction, inflammation, anesthetic effect

## Abstract

**Objective:**

The objective of this article is to make a comparison of the anesthetic effects of the inside and outside fascia iliaca compartment block (FICB) in total hip arthroplasty (THA) and to study the effect of the different approaches of the FICB on postoperative cognitive dysfunction (POCD) and serum inflammatory cytokines in THA patients.

**Methods:**

A total of 60 patients who received THA treatment from January 2021 to December 2021 were divided into two groups, namely, Inside group (inside approach of the FICB) and Outside group (outside approach of the FICB), according to the different approaches of the FICB. Forty-eight hours after surgery, we compared the use of ropacaine dosage, visual analogue scale (VAS) score, the use of patient-controlled analgesia (PCA), mini-mental state examination (MMSE) score, the incidence of POCD, and the serum levels of IL-1, IL-6. Secondary indicators include surgical indicators and the quality of anesthesia cannula.

**Results:**

The ratio of re-fix the catheter, intubation time, and the use of ropacaine dosage at 48 h after surgery in the Outside group were significantly higher than that in the Inside group (*p* < 0.05), while the depth of cannulation in the Outside group was significantly lower than that in the Inside group (*p* < 0.05). VAS scores were comparable between the Inside and the Outside groups, except at 24 h after surgery. The use of PCA from 24 to 48 h after surgery in the Outside group was significantly higher than that in the Inside group (*p* < 0.05). The MMSE score and the incidence of POCD in the Outside group were higher than that in the Inside group. At the same time, the serum IL-1β levels at 1 and 6 h after surgery and the serum IL-6 levels at 1, 6, 24, and 48 h after surgery in the Outside group were significantly higher than that in the Inside group (*p* < 0.05).

**Conclusion:**

Compared with the outside approach of the FICB, the inside approach of the FICB has better anesthetic effect, better postoperative analgesia, fewer postoperative analgesics, lower incidence of POCD, and lower serum cytokines during the treatment of THA patients.

## Introduction

With the aging of the population, the number of patients undergoing hip replacement surgery also increases. The number of patients requiring THA is expected to rise to 6.3 million by 2050 in the United States, and the number of patients in China will undoubtedly be higher (1, 2). THA is a common treatment for patients with hip fractures, but it is traumatic and painful, and the most severe pain occurs within 24 h after surgery (3, 4). Previous studies have shown that if postoperative pain cannot be effectively controlled at the initial stage, the risk of developing chronic pain will increase, which will not only affect the patient’s postoperative recovery, but also have a serious impact on the patient’s long-term quality of life (5, 6). Therefore, effective pain management after THA can not only promote patient recovery and reduce postoperative complications, but also improve patient satisfaction and prognosis. Due to the high proportion of middle-aged and elderly patients receiving THA, avoiding postoperative cognitive dysfunction (POCD) is also a major factor that impacts the choice of the anesthesia method used (7, 8). POCD is a disorder of postoperative brain function in patients without preoperative mental disorders affected by the perioperative period, resulting in different degrees of mental activities such as cognition, emotion, behavior, and will. It can be as high as 25%–50% in hospitalized patients (9, 10). Consistent with postoperative pain, anesthesia is an important factor impacting the occurrence of POCD in patients treated with THA.

Currently, general anesthesia, spinal anesthesia, and nerve block can all be used for THA treatment, and the disadvantages of different anesthesia methods vary (11, 12). General anesthesia can significantly affect the respiratory and circulatory systems, resulting in opioid-related adverse reactions. Severe nausea and vomiting will affect patients’ early eating habit and reduce their levels of satisfaction, and patients with general anesthesia are at a higher risk for POCD (13). Intraspinal anesthesia is also a commonly used anesthesia scheme for THA patients in clinical practice, but the elderly often encounter many problems such as spinal degeneration, ligament calcification, and difficulty in placing the puncture position, which, in turn, increases the difficulty of the puncture condition (14). With the recent advancement of ultrasound visualization technology, nerve block technology is being increasingly used for surgical anesthesia and postoperative analgesia in fracture patients (15, 16). The advantage of nerve block technology is that it can not only help in good pain management during and after surgery, but also help avoid the impact of general anesthesia on cardiopulmonary function, maintain hemodynamic stability, and reduce the risk of POCD (17).

In this study, we compared the anesthetic effects of the inside and outside FICB in total hip arthroplasty (THA) and studied the effects of the different approaches of the FICB on POCD and serum inflammatory cytokines in THA patients. Twenty-four hours after surgery is the most obvious time point for THA patients to feel pain, and this period is a key time point to distinguish the effects of different anesthesia methods on postoperative analgesia.

## Materials and Methods

### Patients and Ethics Statement

A total of 60 patients who received THA treatment from January 2021 to December 2021 were included in the present study. Inclusion criteria: age 18–80 years, American Society of Anesthesiologists (ASA) stages I–III, meeting surgical standards. At the same time, the following types of patients were excluded from this study: those allergic to local anesthetics, those who have peripheral neuropathy, deafness, or language impairment or inability to communicate effectively, those having neurological diseases, a history of alcoholism, and drug dependence, those whose operation time exceeds 3 h or surgical blood loss exceeds 800 mL, those with diabetes, chronic infectious diseases, malignant tumors, organ dysfunction, a mini-mental state examination (MMSE) score <27, and all those patients who are unwilling to participate in this study.

### Anesthesia Protocol

All patients were monitored for vital signs. After the establishment of intravenous access, they were given atropine 0.3 mg and tropisetron 5 mg, and they inhaled pure oxygen by using a face mask, 6 L/min. Anesthesia induction protocol: dexmedetomidine 0.3 µg/kg, sufentanil 0.2–0.4 µg/kg, etomidate 0.1–0.3 mg/kg, cisatracurium 0.2 mg intravenously when the patient’s bispect ral index (BIS) value drops to 55–45/kg, and mechanical ventilation through orotracheal intubation after jaw relaxation. During the operation, the patients underwent a continuous inhalation of 2%–4% sevoflurane, an intermittent bolus injection of cisatracurium, and a continuous pump injection of sufentanil 0.05–0.2 µg/kg/h to maintain the levels of anesthesia. Sevoflurane and sufentanil were discontinued at the start of the surgery.

### Fascia Iliaca Compartment Block Protocol

After the patients were administered general anesthesia with endotracheal intubation, a persistent fascia iliacus space block procedure was performed with an ultrasound-guided inside approach (Inside group) or outside approach (Outside group). After the administration of anesthesia, the patients were placed in a supine position with both legs straight, with natural mild abduction and external rotation. The needle kit contains a 55- mm 18G nerve block catheter kit. The femoral artery, femoral nerve, fascia lata, and iliac fascia were observed under ultrasound guidance. The puncture needle was inserted from the inside or outside of the femur by the in-plane technique, avoiding the femoral artery, and the needle tip reached the fascia iliacus space. A diffusion of normal saline was observed. When the normal saline diffused in a fusiform shape, it indicated that the needle tip was indeed in the fascia iliacus space, and 30 mL of 0.3% ropivacaine was injected. After the iliac fascia space was expanded by the water separation technique, the steel needle was fixed, the trocar was inserted, the steel needle was withdrawn, 1 mL of normal saline was injected into the trocar to ensure the patency of the cannula, and finally, the catheter was inserted.

### Data Collection

We recorded demographics including gender, age, body mass index (BMI), and ASA grade, surgical characteristics such as operation time, intraoperative blood loss, intraoperative urine volume, and intraoperative infusion volume, and nerve block–related indicators such as the ratio of re-fix the catheter, intubation time, the use of ropacaine dosage at 48 h after surgery, the depth of cannulation, and the time of ultrasound and puncture injection.

At 1, 6, 24, and 48 h after THA, the visual analogue scale (VAS) score was used to assess the postoperative pain of patients: VAS scores ranged from 0 to 10, with higher scores indicating greater pain (16, 17). At 6, 24, and 48 h, MMSE was used to assess the postoperative cognitive function of the patients: an MMSE score <27 indicated POCD.

### Statistical Analysis

Data in this study were analyzed by using SPSS 20.0 (NIH, USA). Measurement data that conformed to normal distribution were presented as mean ± standard deviation, and the difference in the measurement data between the two groups was compared using an independent-sample *t*-test. Categorical data were presented as numbers and percentages and examined by using Chi-squared analysis or Fisher’s exact-probability test. A score of *p* < 0.05 was considered statistically significant.

## Results

### Demographic and Surgical Characteristics

In the present study, we included 60 THA patients and divided them into two groups according to the anesthesia method used: Inside group and Outside group. The baseline data of gender, age, BMI, and ASA grade between the two groups were comparable (*p* > 0.05) (**Table 1**). At the same time, the surgical characteristics of operation time, intraoperative blood loss, intraoperative urine volume, and intraoperative infusion volume between the two groups were comparable (*p* > 0.05) (**Table 1**).

**Table 1:** Comparison of demographic and surgical indicators between two groups.

Index	Inside group (*n* = 30)	Outside group (*n* = 30)	*t*/χ^2^	*p*
Gender (*n*)				
Male	14/	15	0.067	0.796
Female	16	15		
Age (years)	59.3 ± 7.8	60.2 ± 9.4	0.503	0.602
BMI (kg/m^2^)	24.5 ± 0.58	24.8 ± 0.74	1.777	0.081
ASA grade				
I + II	19	17	0.278	0.598
III	11	13		
Surgical indicators				
Time (min)	117.1 ± 4.7	116.6 ± 2.31	0.521	0.604
Bleeding volume (mL)	425.5 ± 157.7	427.5 ± 142.1	1.354	0.181
Urine (mL)	390.2 ± 38.9	376.8 ± 37.8	1.360	0.179
Infusion volume (mL)	1,672.6 ± 172.6	1,744.5 ± 215.2	1.426	0.159

### Anesthetic Effect

To compare the effect of anesthesia in the two groups, we found that the ratio of re-fix the catheter, intubation time, and the use of ropacaine dosage at 48 h after surgery in the Outside group were significantly higher than those in the Inside group (*p* < 0.05), while the cannulation depth in the Outside group was significantly lower than that in the Inside group (*p* < 0.05) (**Table 2**). In addition, there was no significant difference between the two groups in regard to the time of ultrasound and puncture injection (*p* > 0.05) (**Table 2**).

**Table 2:** Comparison of nerve block–related indicators between two groups.

Group	Inside group (*n* = 30)	Outside group (*n* = 30)	*t*/χ^2^	*p*
Re-fix the catheter [*n* (%)]	2 (6.7)	11 (36.7)	7.954	0.005
Ultrasound time (s)	75.4 ± 5.9	77.5 ± 8.5	1.107	0.273
Puncture injection time (s)	95.5 ± 6.6	96.6 ± 10.2	0.479	0.621
Intubation time (s)	67.2 ± 4.6	159.0 ± 14.5	33.060	<0.001
Cannulation depth (cm)	11.2 ± 2.3	7.4 ± 2.2	6.613	<0.001
Ropacaine dosage (mL)	250.3 ± 51.8	279.4 ± 49.2	2.232	0.029

**Figure 1:**
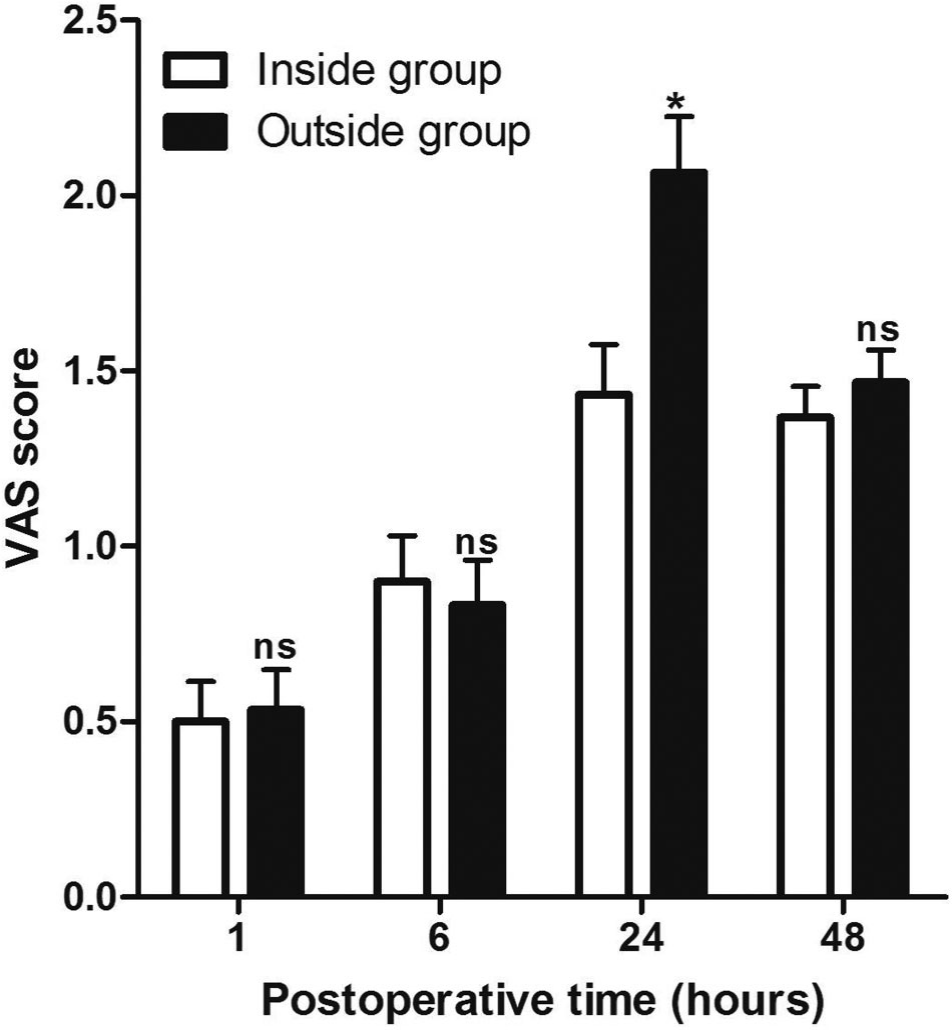
Comparison of visual analogue scale scores at different postoperative times between two groups. Compared with the Inside group, ns *p* > 0.05 and *p < 0.05.

**Figure 2:**
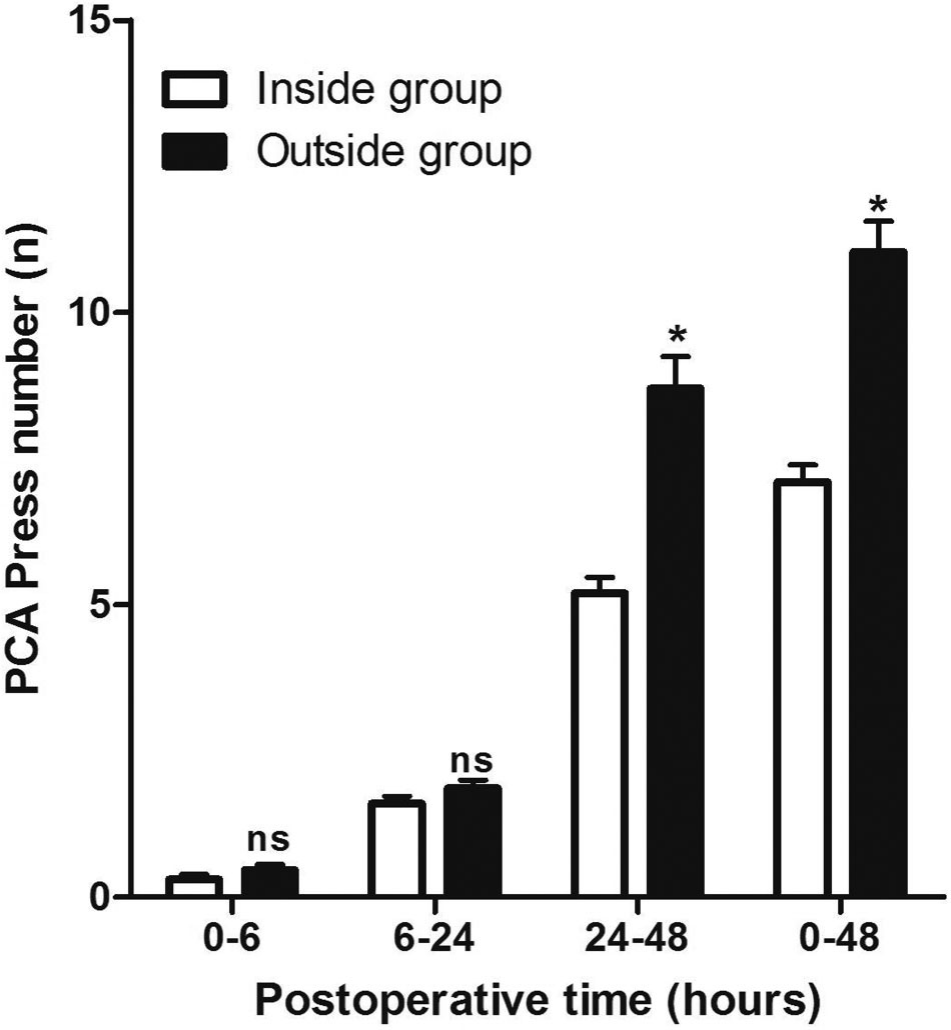
Comparison of patient-controlled analgesia press number at different postoperative times between two groups. Compared with the Inside group, ns *p* > 0.05 and *p < 0.05.

At 1, 6, 24, and 48 h after the operation, we evaluated the pain perception (VAS score) of the patients and recorded the number of times of patient-controlled analgesia (PCA) use within 48 h after the operation. As shown in **Figure 1**, the VAS scores were comparable between the Inside and the Outside groups, except at 1, 6, and 48 h after surgery, while the VAS score in the Inside group at 24 h after surgery was significantly lower than that in the Outside group. At the same time, the use of PCA from 0 to 6 h and from 6 h to 24 h after operation between the two groups was comparable (*p* > 0.05), while the use of PCA from 24 h to 48 h after surgery in the Outside group was significantly higher than that in the Inside group (*p* < 0.05) (**Figure 2**).

### Postoperative cognitive dysfunction

Compared with the Inside group, the MMSE score of patients in the Outside group at 6 h (T1), 24 h (T2), and 48 h (T3) after surgery were significantly decreased (*p* < 0.05) (**Table 3**). At the same time, the incidence of POCD in the Outside group at 6 h, 24 h, and 48 h after surgery were higher than that in the Inside group, but there was no significant difference (*p* > 0.05) (**Table 3**).

**Table 3:** Comparison of mini-mental state examination (MMSE) score and postoperative cognitive dysfunction (POCD) incidence at different postoperative times between two groups.

Group	*n*	MMSE score			POCD [*n* (%)]		
		T1	T2	T3	T1	T2	T3
Inside group	30	29.4 ± 2.9	31.1 ± 2.9	31.3 ± 2.8	2 (6.7)	1 (3.33)	0 (0.0)
Outside group	30	26.8 ± 3.3	27.6 ± 3.5	28.2 ± 2.8	7 (23.3)	5 (16.7)	1 (3.3)
*t*/χ^2^		3.195	2.900	5.348	3.268	2.963	1.017
*p*		0.002	0.005	<0.001	0.071	0.085	0.313

### Serum inflammatory cytokines

**Figure 3:**
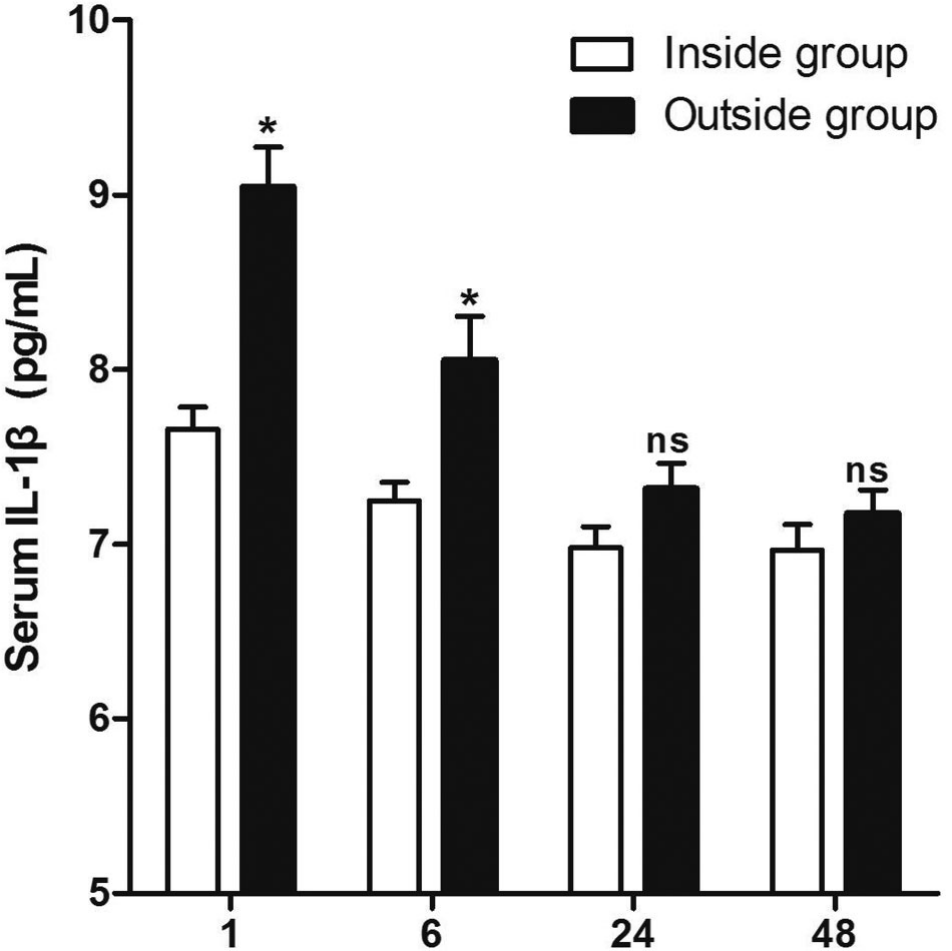
Comparison of serum IL-1β at different postoperative times between two groups. Compared with the Inside group, ns *p* > 0.05 and *p < 0.05.

**Figure 4:**
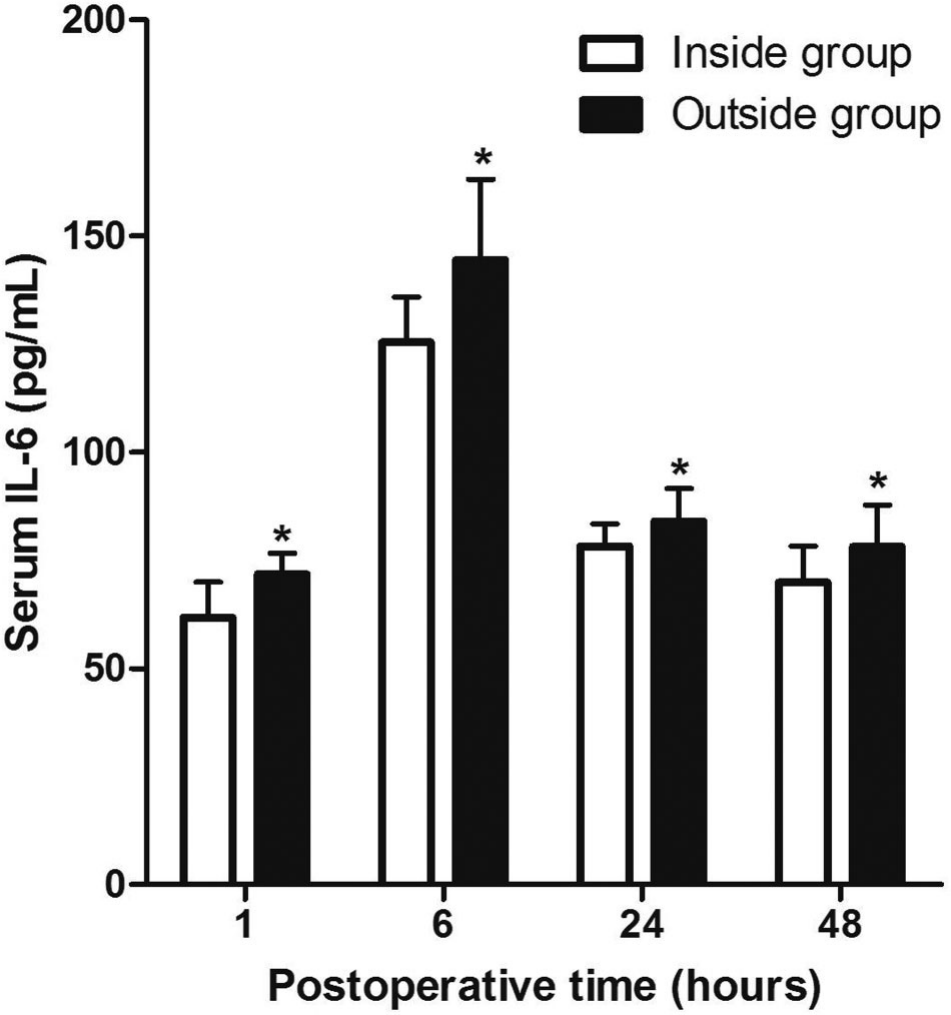
Comparison of serum IL-6 at different postoperative times between two groups. Compared with the Inside group, ns *p* > 0.05 and *p < 0.05.

As shown in **Figure 3**, the serum levels of IL-1β in patients of the Outside group at 1 and 6 h after surgery were significantly higher than those of the Inside group, while there was no significant difference at 24and 48 h after surgery. However, the serum levels of IL-6 in patients of the Outside group after surgery were all significantly higher than those in the Inside group (*p* < 0.05) (**Figure 4**).

## Discussion

THA is one of the common surgeries in clinical joint surgery. In the past, spinal anesthesia was commonly used, but its application was limited due to the blocking of the sympathetic nerve, the fluctuation of hemodynamics, and perioperative anticoagulation therapy (1, 2). At present, general anesthesia, combined with nerve block anesthesia, has been widely used in clinical practice, especially in THA, and its advantages are many, such as providing effective relief for perioperative pain, controlling and reducing the dosage of opioids, and promoting the rapid recovery of patients (13, 14). The iliac fascia is formed by the muscle fascia of the psoas major, iliacus, and pubis muscles, and the iliac fascia space is a potential gap in the anterior iliac fascia and the posterior iliopsoas and iliopsoas fascia (15–17). The three main nerves originating from the lumbar plexus—the femoral nerve, the lateral femoral cutaneous nerve, and the obturator nerve—run behind the fascia iliacus and are located together in the fascia iliacus space.

In this study, all 60 THA patients received general anesthesia, combined with the FICB, and all of them were in agreement with the fact that both the approaches of the FICB are different. We found that the ratio of re-fix the catheter, intubation time, and the use of ropacaine dosage at 48 h after surgery in the Outside group were significantly higher than that in the Inside group, while the cannulation depth in the Outside group was significantly lower than that in the Inside group, which suggested that the inside approach of the FICB was an easier and more effective one in anesthetizing the patients than the outside FICB approach. Needle insertion in the outside FICB approach is directed toward the inside, so that the direction of the inserted catheter is away from the lateral femoral cutaneous nerve, while the needle insertion direction of the inside FICB approach points to the outside, so that the indwelling catheter tip is closer to the lateral femoral cutaneous nerve (18, 19). The lateral femoral cutaneous nerve block ratio was higher with less postoperative analgesia (20–22). The lateral femoral cutaneous nerve divides into anterior and posterior branches at about 5 cm below the anterior superior iliac spine, and the posterior branch moves to the posterior and inferior ear, and distributes to the skin near the greater trochanter of the femur. Therefore, the block rate of the lateral femoral cutaneous nerve is higher, and there is lesser use of PCA and ropivacaine consumption at 48 h postoperatively (20–22).

In addition, we found that the VAS score of patients in the Outside group was significantly higher than that in the Inside group at 24 h postoperatively, and the use of PCA from 24 to 48 h after surgery in the Outside group was significantly higher than that in the Inside group, which suggested that the postoperative analgesia effect of the inside FICB approach is better than the outside approach in THA patients. At the same time, we also found that the MMSE score in the Outside group was lower than that in the Inside group at 6, 24, and 48 h postoperatively, while the incidence of POCD in the Outside group was higher than that in the Inside group at 6, 24, and 48 h postoperatively. Anesthesia protocol is an important factor impacting postoperative pain and the incidence of POCD (23, 24). Our results have shown that the inside approach of the FICB was more effective in anesthetizing patients than the outside approach.

Surgery activates the immune system of patients to produce a strong peripheral inflammatory response. The type of surgery and surgical trauma will affect the level of serum inflammatory factors. Peripheral inflammatory factors can cause central nervous system (CNS) inflammatory response through direct or indirect pathways. When excessive CNS inflammatory response occurs, it can affect cognitive function by producing the following effects (25–30). First, inflammatory cytokines affect neural activity and synaptic connections (25). Second, high concentrations of inflammatory factors can produce neurotoxicity and cause neurodegeneration, resulting in impaired cognitive function (26, 27). Third, high levels of inflammatory cytokines cause nerve damage in the hippocampus (28). Last, inflammatory factors can stimulate actin in cells other than neurons in the brain, resulting in actin regeneration, a change that plays an important role in neurodegeneration (29, 30). Previous studies had showed that postoperative serum inflammatory cytokines were elevated in THA patients, and serum IL-1β and IL-6 levels were associated with the occurrence of POCD (31, 32). In the present study, the serum levels of IL-1β in patients of the Outside group at 1 and 6 h after surgery are significantly higher than those in the Inside group, and the serum levels of IL-6 in patients of the Outside group after surgery are significantly higher than those in the Inside group.

However, there were several limitations to our study. First, this study focused only on the short-term pain and POCD of THA patients post surgery and lacked a comparison of the long-term efficacy of THA patients. Although we found differences in postoperative serum inflammatory cytokine levels in THA patients between the two approaches of the FICB, we could not further study their effects on POCD due to the inclusion of the limited sample size.

## Conclusion

Compared with the outside approach of the FICB, the inside FICB approach has better anesthetic effect, better postoperative analgesia, fewer postoperative analgesics, lower incidence of POCD, and lower serum cytokines during the treatment of THA patients.

## Data Availability

The original contributions presented in the study are included in the article/supplementary material, and further inquiries can be directed to the corresponding author.
